# Bis(2-amino­benzothia­zol-3-ium) bis­(7-oxabicyclo­[2.2.1]heptane-2,3-dicarboxyl­ato-κ^3^ *O* ^2^,*O* ^3^,*O* ^7^)zincate hexa­hydrate

**DOI:** 10.1107/S1600536812017886

**Published:** 2012-04-28

**Authors:** Fan Zhang, Tian-Xi Lv, Jie Feng, Qiu-Yue Lin

**Affiliations:** a Zhejiang Key Laboratory for Reactive Chemistry on Solid Surfaces, Institute of Physical Chemistry, Zhejiang Normal University, Jinhua, Zhejiang 321004, People’s Republic of China; b College of Chemistry and Life Science, Zhejiang Normal University, Jinhua 321004, Zhejiang, People’s Republic of China

## Abstract

In the title hydrated mol­ecular salt, (C_7_H_7_N_2_S)_2_[Zn(C_8_H_8_O_5_)_2_]·6H_2_O, which is isotypic with its Mn^II^, Co^II^ and Ni^II^ analogues, the Zn^2+^ ion lies on a crystallographic inversion centre and a distorted ZnO_6_ octa­hedral coordination geometry arises from the two doubly deprotonated *O*,*O*′,*O*′′-tridentate ligands. In the crystal, the components are linked by N—H⋯O_a_, N—H⋯O_w_, O_w_—H⋯O_a_ and O_w_—H⋯O_w_ hydrogen bonds (w = water and a = anion).

## Related literature

For background to the applications of norcantharidin (systematic name: 7-oxabicyclo­[2,2,1]heptane-2,3-dicarb­oxy­lic anhydride), see: Zeng & Lu (2006 ▶). For the isotypic Mn^II^, Co^II^ and Ni^II^ structures, see: Wang *et al.* (2010*a* ▶,*b* ▶, 2012 ▶).
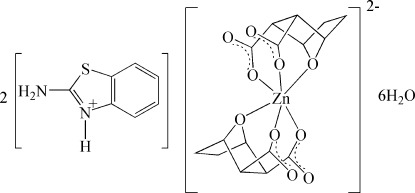


## Experimental

### 

#### Crystal data

(C_7_H_7_N_2_S)_2_[Zn(C_8_H_8_O_5_)_2_]·6H_2_O
*M* *_r_* = 844.21
Triclinic, 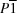

*a* = 6.6983 (7) Å
*b* = 10.1497 (11) Å
*c* = 13.2082 (14) Å
α = 90.172 (7)°
β = 91.097 (7)°
γ = 99.251 (7)°
*V* = 886.11 (16) Å^3^
*Z* = 1
Mo *K*α radiation
μ = 0.89 mm^−1^
*T* = 296 K
0.12 × 0.08 × 0.06 mm

#### Data collection

Bruker APEXII CCD diffractometer
Absorption correction: multi-scan (*SADABS*; Sheldrick, 1996 ▶) *T*_min_ = 0.914, *T*_max_ = 0.951
11657 measured reflections
3108 independent reflections
1839 reflections with *I* > 2σ(*I*)
*R*_int_ = 0.228

#### Refinement

*R*[*F* ^2^ > 2σ(*F* ^2^)] = 0.047
*wR*(*F* ^2^) = 0.087
*S* = 0.91
3108 reflections
241 parameters
9 restraints
H-atom parameters constrained
Δρ_max_ = 0.48 e Å^−3^
Δρ_min_ = −0.74 e Å^−3^

### 

Data collection: *APEX2* (Bruker, 2006 ▶); cell refinement: *SAINT* (Bruker, 2006 ▶); data reduction: *SAINT*; program(s) used to solve structure: *SHELXS97* (Sheldrick, 2008 ▶); program(s) used to refine structure: *SHELXL97* (Sheldrick, 2008 ▶); molecular graphics: *SHELXTL* (Sheldrick, 2008 ▶); software used to prepare material for publication: *SHELXL97*.

## Supplementary Material

Crystal structure: contains datablock(s) I, global. DOI: 10.1107/S1600536812017886/hb6746sup1.cif

Structure factors: contains datablock(s) I. DOI: 10.1107/S1600536812017886/hb6746Isup2.hkl

Additional supplementary materials:  crystallographic information; 3D view; checkCIF report

## Figures and Tables

**Table 1:** Selected bond lengths (Å)

Zn1—O1	2.014 (2)
Zn1—O3	2.132 (2)
Zn1—O5	2.176 (3)

**Table 2:** Hydrogen-bond geometry (Å, °)

*D*—H⋯*A*	*D*—H	H⋯*A*	*D*⋯*A*	*D*—H⋯*A*
N1—H1*A*⋯O4^i^	0.86	1.82	2.675 (4)	173
N2—H2*A*⋯O3^i^	0.86	2.00	2.853 (4)	172
N2—H2*B*⋯O2*W*^ii^	0.86	2.02	2.838 (4)	158
O1*W*—H1*WA*⋯O4	0.85	2.01	2.818 (3)	160
O1*W*—H1*WB*⋯O2*W*	0.85	1.95	2.793 (4)	170
O2*W*—H2*WA*⋯O2	0.85	1.85	2.683 (3)	167
O2*W*—H2*WB*⋯O3*W*	0.85	1.92	2.765 (3)	170
O3*W*—H3*WA*⋯O1*W*^ii^	0.85	2.21	3.024 (3)	160
O3*W*—H3*WB*⋯O1*W*^iii^	0.85	2.00	2.793 (4)	156

Symmetry codes: (i) 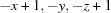; (ii) 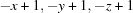; (iii) 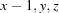.

**Fig. 1.**
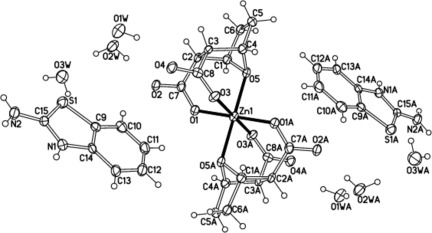
A view of (I) showing displacement ellipsoids drawn at the 30% probability level. Atoms with label suffix A are generatd by (1–x, –y, –z).

## supplementary crystallographic information

### Comment

7-oxabicyclo[2,2,1]heptane-2,3-dicarboxylic anhydride (norcantharidin), as a traditional Chinese drug, has great anti-cancer activity. (Zeng *et al.*, 2006). A isostructural manganese complex (Wang *et al.*, 2010*a*) and a cobalt complex (Wang *et al.*, 2010*b*) has been reported. The molecular structure of the title complex is shown in Fig.1. The zinc atom is six-coordinated in a distorted octahedral coordination mode, binding to two bridging O atoms of the bicycloheptane unit and four carboxylate O atoms of two symmetry-related and fully deprotonated ligands. 2-aminobenzothiazole don't involved the coordination, and N atom of thiazole ring is protonated.The crystal structure is stabilized by N—H···O hydrogen-bonding interactions between the cations and anions and O—H···O hydrogen bonds including the crystal water molecules.

### Experimental

A mixture of 0.5 mmol norcantharidin, 0.5 mmol zinc acetate, 0.5 mmol 2-aminobenzothiazole and 15 mL distilled water was sealed in a 25 mL Teflon-lined stainless vessel and heated at 443 K for 3 d, then cooled slowly to room temperature. The solution was filtered and colourless blocks were recovered.

### Refinement

H atoms bonded to C atoms were positioned geometrically and refined using a riding model [aliphatic of tertiary carbon C—H = 0.98 Å, aliphatic of secondary carbon C—H = 0.97 Å, N—H = 0.86 Å, both with *U*_iso_(H) = 1.2*U*_eq_(C)]. The H atoms bonded to O atoms were located in a difference Fourier maps and refined with O—H distance restraints of 0.85 (4) Å and *U*_iso_(H) = 1.5*U*_eq_(O).

### Crystal data

**Table:**

(C_7_H_7_N_2_S)_2_[Zn(C_8_H_8_O_5_)_2_]·6H_2_O	*Z* = 1
*M**_r_* = 844.21	*F*(000) = 440
Triclinic, *P*1	*D*_x_ = 1.582 Mg m^−^^3^
Hall symbol: -P 1	Mo *K*α radiation, λ = 0.71073 Å
*a* = 6.6983 (7) Å	Cell parameters from 2505 reflections
*b* = 10.1497 (11) Å	θ = 1.5–25.0°
*c* = 13.2082 (14) Å	µ = 0.89 mm^−^^1^
α = 90.172 (7)°	*T* = 296 K
β = 91.097 (7)°	Block, colorless
γ = 99.251 (7)°	0.12 × 0.08 × 0.06 mm
*V* = 886.11 (16) Å^3^	

### Data collection

**Table:**

Bruker APEXII CCD diffractometer	3108 independent reflections
Radiation source: fine-focus sealed tube	1839 reflections with *I* > 2σ(*I*)
Graphite monochromator	*R*_int_ = 0.228
ω scans	θ_max_ = 25.0°, θ_min_ = 1.5°
Absorption correction: multi-scan (*SADABS*; Sheldrick, 1996)	*h* = −7→7
*T*_min_ = 0.914, *T*_max_ = 0.951	*k* = −12→12
11657 measured reflections	*l* = −15→14

### Refinement

**Table:**

Refinement on *F*^2^	Primary atom site location: structure-invariant direct methods
Least-squares matrix: full	Secondary atom site location: difference Fourier map
*R*[*F*^2^ > 2σ(*F*^2^)] = 0.047	Hydrogen site location: inferred from neighbouring sites
*wR*(*F*^2^) = 0.087	H-atom parameters constrained
*S* = 0.91	*w* = 1/[σ^2^(*F*_o_^2^) + (0.0123*P*)^2^] where *P* = (*F*_o_^2^ + 2*F*_c_^2^)/3
3108 reflections	(Δ/σ)_max_ < 0.001
241 parameters	Δρ_max_ = 0.48 e Å^−^^3^
9 restraints	Δρ_min_ = −0.74 e Å^−^^3^

### Special details

**Table:**

Geometry. All e.s.d.'s (except the e.s.d. in the dihedral angle between two l.s. planes) are estimated using the full covariance matrix. The cell e.s.d.'s are taken into account individually in the estimation of e.s.d.'s in distances, angles and torsion angles; correlations between e.s.d.'s in cell parameters are only used when they are defined by crystal symmetry. An approximate (isotropic) treatment of cell e.s.d.'s is used for estimating e.s.d.'s involving l.s. planes.
Refinement. Refinement of *F*^2^ against ALL reflections. The weighted *R*-factor *wR* and goodness of fit *S* are based on *F*^2^, conventional *R*-factors *R* are based on *F*, with *F* set to zero for negative *F*^2^. The threshold expression of *F*^2^ > σ(*F*^2^) is used only for calculating *R*-factors(gt) *etc*. and is not relevant to the choice of reflections for refinement. *R*-factors based on *F*^2^ are statistically about twice as large as those based on *F*, and *R*- factors based on ALL data will be even larger.

### Fractional atomic coordinates and isotropic or equivalent isotropic displacement parameters (Å^2^)

**Table:**

	*x*	*y*	*z*	*U*_iso_*/*U*_eq_	
Zn1	0.5000	0.0000	0.0000	0.0324 (2)	
S1	0.32760 (16)	0.26730 (12)	0.52749 (7)	0.0407 (3)	
O1	0.3674 (4)	0.1456 (3)	0.06056 (17)	0.0375 (8)	
O1W	0.8090 (4)	0.3956 (3)	0.37387 (17)	0.0545 (9)	
H1WA	0.8277	0.3325	0.3345	0.082*	
H1WB	0.7307	0.4430	0.3454	0.082*	
H2WA	0.4603	0.4745	0.2298	0.082*	
H2WB	0.4268	0.5366	0.3202	0.082*	
H3WA	0.1928	0.5407	0.4659	0.082*	
H3WB	0.0922	0.4941	0.3767	0.082*	
O2	0.3636 (4)	0.3401 (3)	0.13832 (18)	0.0398 (8)	
O2W	0.5164 (4)	0.5222 (3)	0.27869 (18)	0.0509 (9)	
O3	0.6904 (4)	0.0121 (3)	0.13199 (17)	0.0372 (8)	
O3W	0.1943 (4)	0.5453 (3)	0.40167 (19)	0.0681 (11)	
O4	0.7729 (4)	0.1592 (3)	0.25795 (16)	0.0391 (8)	
O5	0.7162 (3)	0.1550 (3)	−0.06915 (16)	0.0318 (8)	
N1	0.2772 (4)	0.0314 (3)	0.6011 (2)	0.0314 (9)	
H1A	0.2680	−0.0334	0.6434	0.038*	
N2	0.3461 (4)	0.1984 (3)	0.7235 (2)	0.0407 (10)	
H2A	0.3394	0.1405	0.7712	0.049*	
H2B	0.3717	0.2822	0.7379	0.049*	
C6	0.8584 (6)	0.3726 (4)	−0.1096 (2)	0.0353 (11)	
H6A	0.8806	0.4636	−0.0841	0.042*	
H6B	0.8494	0.3736	−0.1829	0.042*	
C5	1.0257 (5)	0.2946 (4)	−0.0715 (3)	0.0360 (11)	
H5A	1.0917	0.2585	−0.1276	0.043*	
H5B	1.1265	0.3507	−0.0300	0.043*	
C1	0.6712 (5)	0.2907 (4)	−0.0644 (2)	0.0318 (11)	
H1B	0.5458	0.3011	−0.1008	0.038*	
C4	0.9066 (5)	0.1842 (4)	−0.0096 (3)	0.0313 (11)	
H4A	0.9750	0.1065	−0.0007	0.038*	
C2	0.6616 (5)	0.3148 (4)	0.0498 (2)	0.0284 (10)	
H2C	0.6979	0.4103	0.0652	0.034*	
C3	0.8342 (5)	0.2369 (4)	0.0902 (2)	0.0281 (11)	
H3A	0.9440	0.3005	0.1213	0.034*	
C7	0.4500 (6)	0.2624 (4)	0.0880 (3)	0.0326 (11)	
C8	0.7611 (5)	0.1281 (4)	0.1656 (3)	0.0299 (10)	
C9	0.2753 (5)	0.1307 (4)	0.4436 (3)	0.0335 (11)	
C10	0.2575 (5)	0.1337 (4)	0.3394 (3)	0.0407 (12)	
H10A	0.2714	0.2138	0.3043	0.049*	
C11	0.2178 (6)	0.0109 (5)	0.2894 (3)	0.0468 (14)	
H11A	0.2094	0.0088	0.2190	0.056*	
C12	0.1909 (6)	−0.1066 (5)	0.3418 (3)	0.0467 (14)	
H12A	0.1586	−0.1865	0.3061	0.056*	
C13	0.2104 (5)	−0.1101 (4)	0.4473 (3)	0.0386 (12)	
H13A	0.1969	−0.1901	0.4826	0.046*	
C14	0.2511 (5)	0.0122 (4)	0.4961 (3)	0.0293 (11)	
C15	0.3173 (5)	0.1590 (4)	0.6289 (3)	0.0304 (11)	

### Atomic displacement parameters (Å^2^)

**Table:**

	*U*^11^	*U*^22^	*U*^33^	*U*^12^	*U*^13^	*U*^23^
Zn1	0.0350 (4)	0.0305 (5)	0.0304 (4)	0.0014 (4)	−0.0001 (3)	−0.0092 (3)
S1	0.0497 (7)	0.0374 (9)	0.0338 (6)	0.0033 (6)	0.0004 (5)	−0.0002 (6)
O1	0.0345 (16)	0.038 (2)	0.0393 (16)	0.0037 (15)	0.0032 (12)	−0.0124 (15)
O1W	0.066 (2)	0.045 (2)	0.0543 (19)	0.0145 (18)	−0.0097 (15)	−0.0099 (17)
O2	0.0357 (16)	0.038 (2)	0.0468 (17)	0.0079 (15)	0.0043 (13)	−0.0186 (15)
O2W	0.056 (2)	0.041 (2)	0.0537 (18)	0.0038 (17)	−0.0062 (14)	−0.0156 (17)
O3	0.0510 (19)	0.027 (2)	0.0307 (15)	−0.0005 (16)	−0.0063 (13)	−0.0028 (14)
O3W	0.063 (2)	0.083 (3)	0.053 (2)	−0.002 (2)	0.0003 (16)	−0.014 (2)
O4	0.0530 (19)	0.037 (2)	0.0254 (15)	0.0015 (16)	−0.0020 (13)	−0.0036 (14)
O5	0.0363 (16)	0.032 (2)	0.0266 (14)	0.0046 (15)	0.0020 (12)	−0.0110 (14)
N1	0.0330 (19)	0.033 (3)	0.0284 (18)	0.0064 (18)	0.0029 (14)	0.0008 (17)
N2	0.055 (2)	0.036 (3)	0.0313 (19)	0.007 (2)	0.0034 (16)	−0.0062 (18)
C6	0.051 (3)	0.026 (3)	0.028 (2)	0.004 (2)	0.0034 (19)	−0.003 (2)
C5	0.039 (3)	0.035 (3)	0.032 (2)	0.000 (2)	0.0097 (18)	−0.002 (2)
C1	0.034 (2)	0.031 (3)	0.031 (2)	0.005 (2)	−0.0005 (18)	−0.003 (2)
C4	0.027 (2)	0.030 (3)	0.037 (2)	0.008 (2)	−0.0026 (18)	−0.005 (2)
C2	0.036 (2)	0.021 (3)	0.028 (2)	0.005 (2)	0.0018 (17)	−0.0092 (19)
C3	0.025 (2)	0.028 (3)	0.029 (2)	0.001 (2)	−0.0013 (17)	−0.011 (2)
C7	0.031 (2)	0.043 (3)	0.024 (2)	0.010 (2)	−0.0052 (17)	−0.007 (2)
C8	0.025 (2)	0.031 (3)	0.034 (2)	0.007 (2)	−0.0009 (18)	0.008 (2)
C9	0.024 (2)	0.043 (3)	0.032 (2)	0.001 (2)	0.0043 (18)	−0.004 (2)
C10	0.039 (3)	0.049 (4)	0.034 (2)	0.007 (2)	0.0040 (19)	0.007 (2)
C11	0.042 (3)	0.070 (4)	0.026 (2)	0.002 (3)	0.004 (2)	−0.008 (3)
C12	0.033 (3)	0.061 (4)	0.046 (3)	0.006 (3)	0.000 (2)	−0.024 (3)
C13	0.029 (2)	0.048 (3)	0.039 (2)	0.008 (2)	0.0051 (18)	−0.012 (2)
C14	0.023 (2)	0.042 (3)	0.023 (2)	0.005 (2)	0.0062 (16)	−0.002 (2)
C15	0.028 (2)	0.030 (3)	0.033 (2)	0.003 (2)	0.0066 (18)	−0.006 (2)

### Geometric parameters (Å, º)

**Table:**

Zn1—O1	2.014 (2)	C6—C1	1.521 (5)
Zn1—O1^i^	2.014 (2)	C6—C5	1.550 (5)
Zn1—O3^i^	2.132 (2)	C6—H6A	0.9700
Zn1—O3	2.132 (2)	C6—H6B	0.9700
Zn1—O5^i^	2.176 (3)	C5—C4	1.518 (5)
Zn1—O5	2.176 (3)	C5—H5A	0.9700
S1—C15	1.731 (4)	C5—H5B	0.9700
S1—C9	1.759 (4)	C1—C2	1.532 (4)
O1—C7	1.274 (4)	C1—H1B	0.9800
O1W—H1WA	0.8499	C4—C3	1.536 (4)
O1W—H1WB	0.8500	C4—H4A	0.9800
O2—C7	1.248 (3)	C2—C7	1.527 (5)
O2W—H2WA	0.8499	C2—C3	1.587 (5)
O2W—H2WB	0.8501	C2—H2C	0.9800
O3—C8	1.272 (4)	C3—C8	1.517 (5)
O3W—H3WA	0.8504	C3—H3A	0.9800
O3W—H3WB	0.8502	C9—C14	1.379 (5)
O4—C8	1.257 (3)	C9—C10	1.379 (4)
O5—C1	1.458 (4)	C10—C11	1.393 (5)
O5—C4	1.473 (4)	C10—H10A	0.9300
N1—C15	1.329 (4)	C11—C12	1.370 (5)
N1—C14	1.403 (4)	C11—H11A	0.9300
N1—H1A	0.8600	C12—C13	1.398 (5)
N2—C15	1.312 (4)	C12—H12A	0.9300
N2—H2A	0.8600	C13—C14	1.382 (5)
N2—H2B	0.8600	C13—H13A	0.9300
			
O1—Zn1—O1^i^	180.0	C2—C1—H1B	113.1
O1—Zn1—O3^i^	92.14 (9)	O5—C4—C5	101.4 (3)
O1^i^—Zn1—O3^i^	87.86 (9)	O5—C4—C3	101.8 (3)
O1—Zn1—O3	87.86 (9)	C5—C4—C3	112.1 (3)
O1^i^—Zn1—O3	92.14 (9)	O5—C4—H4A	113.4
O3^i^—Zn1—O3	180.0	C5—C4—H4A	113.4
O1—Zn1—O5^i^	91.98 (9)	C3—C4—H4A	113.4
O1^i^—Zn1—O5^i^	88.02 (9)	C7—C2—C1	110.4 (3)
O3^i^—Zn1—O5^i^	89.22 (10)	C7—C2—C3	115.0 (3)
O3—Zn1—O5^i^	90.78 (10)	C1—C2—C3	100.7 (2)
O1—Zn1—O5	88.02 (9)	C7—C2—H2C	110.1
O1^i^—Zn1—O5	91.98 (9)	C1—C2—H2C	110.1
O3^i^—Zn1—O5	90.78 (10)	C3—C2—H2C	110.1
O3—Zn1—O5	89.22 (10)	C8—C3—C4	113.7 (3)
O5^i^—Zn1—O5	180.0	C8—C3—C2	113.7 (3)
C15—S1—C9	90.10 (19)	C4—C3—C2	101.0 (3)
C7—O1—Zn1	128.1 (2)	C8—C3—H3A	109.4
H1WA—O1W—H1WB	110.0	C4—C3—H3A	109.4
H2WA—O2W—H2WB	109.4	C2—C3—H3A	109.4
C8—O3—Zn1	117.2 (3)	O2—C7—O1	124.0 (4)
H3WA—O3W—H3WB	109.6	O2—C7—C2	117.8 (4)
C1—O5—C4	95.2 (3)	O1—C7—C2	118.0 (3)
C1—O5—Zn1	116.69 (19)	O4—C8—O3	124.0 (4)
C4—O5—Zn1	112.0 (2)	O4—C8—C3	117.5 (3)
C15—N1—C14	113.7 (3)	O3—C8—C3	118.5 (3)
C15—N1—H1A	123.1	C14—C9—C10	121.7 (4)
C14—N1—H1A	123.1	C14—C9—S1	110.6 (3)
C15—N2—H2A	120.0	C10—C9—S1	127.7 (4)
C15—N2—H2B	120.0	C9—C10—C11	116.9 (4)
H2A—N2—H2B	120.0	C9—C10—H10A	121.6
C1—C6—C5	101.2 (3)	C11—C10—H10A	121.6
C1—C6—H6A	111.5	C12—C11—C10	121.3 (4)
C5—C6—H6A	111.5	C12—C11—H11A	119.4
C1—C6—H6B	111.5	C10—C11—H11A	119.4
C5—C6—H6B	111.5	C11—C12—C13	122.0 (4)
H6A—C6—H6B	109.4	C11—C12—H12A	119.0
C4—C5—C6	102.2 (3)	C13—C12—H12A	119.0
C4—C5—H5A	111.3	C14—C13—C12	116.1 (4)
C6—C5—H5A	111.3	C14—C13—H13A	121.9
C4—C5—H5B	111.3	C12—C13—H13A	121.9
C6—C5—H5B	111.3	C9—C14—C13	121.9 (4)
H5A—C5—H5B	109.2	C9—C14—N1	112.6 (3)
O5—C1—C6	102.7 (3)	C13—C14—N1	125.4 (4)
O5—C1—C2	102.4 (3)	N2—C15—N1	123.4 (4)
C6—C1—C2	111.6 (3)	N2—C15—S1	123.7 (3)
O5—C1—H1B	113.1	N1—C15—S1	113.0 (3)
C6—C1—H1B	113.1		
			
O3^i^—Zn1—O1—C7	−122.1 (3)	C5—C4—C3—C2	72.1 (4)
O3—Zn1—O1—C7	57.9 (3)	C7—C2—C3—C8	−3.6 (4)
O5^i^—Zn1—O1—C7	148.6 (3)	C1—C2—C3—C8	−122.3 (3)
O5—Zn1—O1—C7	−31.4 (3)	C7—C2—C3—C4	118.6 (3)
O1—Zn1—O3—C8	−42.4 (3)	C1—C2—C3—C4	−0.1 (3)
O1^i^—Zn1—O3—C8	137.6 (3)	Zn1—O1—C7—O2	−169.1 (3)
O5^i^—Zn1—O3—C8	−134.3 (2)	Zn1—O1—C7—C2	16.1 (5)
O5—Zn1—O3—C8	45.7 (2)	C1—C2—C7—O2	−127.5 (4)
O1—Zn1—O5—C1	−10.4 (2)	C3—C2—C7—O2	119.3 (4)
O1^i^—Zn1—O5—C1	169.6 (2)	C1—C2—C7—O1	47.6 (5)
O3^i^—Zn1—O5—C1	81.7 (2)	C3—C2—C7—O1	−65.6 (4)
O3—Zn1—O5—C1	−98.3 (2)	Zn1—O3—C8—O4	138.6 (3)
O1—Zn1—O5—C4	97.84 (19)	Zn1—O3—C8—C3	−40.8 (4)
O1^i^—Zn1—O5—C4	−82.16 (19)	C4—C3—C8—O4	153.4 (3)
O3^i^—Zn1—O5—C4	−170.05 (19)	C2—C3—C8—O4	−91.7 (4)
O3—Zn1—O5—C4	9.95 (19)	C4—C3—C8—O3	−27.2 (5)
C1—C6—C5—C4	−1.3 (3)	C2—C3—C8—O3	87.7 (4)
C4—O5—C1—C6	57.0 (3)	C15—S1—C9—C14	0.6 (3)
Zn1—O5—C1—C6	174.86 (19)	C15—S1—C9—C10	−179.7 (3)
C4—O5—C1—C2	−58.8 (3)	C14—C9—C10—C11	−1.2 (5)
Zn1—O5—C1—C2	59.0 (3)	S1—C9—C10—C11	179.2 (3)
C5—C6—C1—O5	−34.6 (3)	C9—C10—C11—C12	2.2 (6)
C5—C6—C1—C2	74.5 (3)	C10—C11—C12—C13	−2.9 (6)
C1—O5—C4—C5	−57.3 (3)	C11—C12—C13—C14	2.3 (5)
Zn1—O5—C4—C5	−178.88 (18)	C10—C9—C14—C13	0.9 (6)
C1—O5—C4—C3	58.4 (3)	S1—C9—C14—C13	−179.4 (3)
Zn1—O5—C4—C3	−63.2 (3)	C10—C9—C14—N1	179.5 (3)
C6—C5—C4—O5	36.1 (3)	S1—C9—C14—N1	−0.9 (4)
C6—C5—C4—C3	−71.8 (3)	C12—C13—C14—C9	−1.4 (5)
O5—C1—C2—C7	−85.8 (3)	C12—C13—C14—N1	−179.7 (3)
C6—C1—C2—C7	165.0 (3)	C15—N1—C14—C9	0.8 (4)
O5—C1—C2—C3	36.2 (3)	C15—N1—C14—C13	179.3 (3)
C6—C1—C2—C3	−73.0 (4)	C14—N1—C15—N2	179.8 (3)
O5—C4—C3—C8	86.7 (4)	C14—N1—C15—S1	−0.3 (4)
C5—C4—C3—C8	−165.7 (3)	C9—S1—C15—N2	179.8 (3)
O5—C4—C3—C2	−35.5 (4)	C9—S1—C15—N1	−0.2 (3)

Symmetry code: (i) −*x*+1, −*y*, −*z*.

### Hydrogen-bond geometry (Å, º)

**Table:**

*D*—H···*A*	*D*—H	H···*A*	*D*···*A*	*D*—H···*A*
N1—H1*A*···O4^ii^	0.86	1.82	2.675 (4)	173
N2—H2*A*···O3^ii^	0.86	2.00	2.853 (4)	172
N2—H2*B*···O2*W*^iii^	0.86	2.02	2.838 (4)	158
O1*W*—H1*WA*···O4	0.85	2.01	2.818 (3)	160
O1*W*—H1*WB*···O2*W*	0.85	1.95	2.793 (4)	170
O2*W*—H2*WA*···O2	0.85	1.85	2.683 (3)	167
O2*W*—H2*WB*···O3*W*	0.85	1.92	2.765 (3)	170
O3*W*—H3*WA*···O1*W*^iii^	0.85	2.21	3.024 (3)	160
O3*W*—H3*WB*···O1*W*^iv^	0.85	2.00	2.793 (4)	156

Symmetry codes: (ii) −*x*+1, −*y*, −*z*+1; (iii) −*x*+1, −*y*+1, −*z*+1; (iv) *x*−1, *y*, *z*.
